# Significance of endolymphatic sac surgery with and/or without simultaneous cochlea implant surgery in respect of vertigo control and speech perception in patients with Menière’s disease

**DOI:** 10.1007/s00405-023-08122-6

**Published:** 2023-07-20

**Authors:** Jennifer L. Spiegel, Bernhard G. Weiss, Joachim Mueller, John-Martin Hempel, Tobias Rader, Mattis Bertlich, Martin Canis, Friedrich Ihler

**Affiliations:** 1 Department for Otorhinolaryngology, University Hospital, Ludwig-Maximilians-Universität München, Marchioninistr. 15, 81377 Munich, Germany; 2 German Center for Vertigo and Balance Disorders, University Hospital, Ludwig-Maximilians-Universität München, Marchioninistr. 15, 81377 Munich, Germany; 3 Department of Dermatology, University Hospital, Ludwig-Maximilians University of Munich, Munich, Germany; 4 Department of Otorhinolaryngology, Head and Neck Surgery, University Medical Center Greifswald, University of Greifswald, Walther-Rathenau-Straße 42, 17489 Greifswald, Germany

**Keywords:** Endolymphatic sac surgery, Cochlear implant, CI, Menière’s disease, MD

## Abstract

**Purpose:**

The focus on treating patients with Menière’s Disease (MD) lies on the reduction of vertigo attacks and the preservation of sensory function. Endolympathic hydrops is considered as an epiphenomenon in MD, which can potentially be altered by endolymphatic sac surgery (ESS). Purpose of the study was to investigate the influences on vertigo control through manipulation of the perilymphatic system with or without ESS.

**Methods:**

Retrospective data analysis of 86 consecutive patients with MD according to current diagnostic criteria after endolymphatic sac surgery alone (ESS^alone^; *n = *45), cochlear implantation (CI) alone (CI^alone^; *n = *12), and ESS with CI (ESS + CI; *n = *29), treated at a tertiary referral center. Main Outcome Measures: vertigo control, speech perception pre- and postoperatively.

**Results:**

Gender, side, and preoperative treatment were similar in all groups. Age was younger in the ESS^alone^-group with 56.2 ± 13.0 years (CI^alone^ = 64.2 ± 11.4 years; ESS + CI = 63.1 ± 9.7 years). Definitive MD was present in all the CI^alone^, in 79.3% of the ESS + CI and in 59.6% of the ESS^alone^-patients. Likewise, vertigo control rate was 100% in the CI^alone^, 89.7% in the ESS + CI and 66.0% in the ESS^alone^-group.

**Conclusions:**

Vertigo control was improved in all three groups, however, superior in groups treated with CI, potentially contributed by the manipulation of both the endo- and perilymphatic systems. A more systematic characterization of the patients with larger case numbers and documentation of follow up data would be needed to evaluate a clinical effect more properly.

## Introduction

Management of patients with Menière’s Disease (MD) has been challenging since the discovery of the disease. An endolymphatic hydrops is hypothesized as an epiphenomenon in MD [1]. It has been reported that colorful variable presentation of symptoms leads to challenges in diagnosing MD, whereupon diagnostic criteria have been established in the 1970s and revised multiple times throughout the decades [2–4]. The most current revision by an international joint effort of the *International Classification of Vestibular Disorders* 2015 defines two categories: definitive MD and probable MD [5]. However, not all MD patients seem to fit into the categories given by these criteria [6], which might be a result of considering solely clinical characteristics for defining this disorder. In addition, providing an individualized treatment concept bears another challenge in the management of MD patients. Throughout the different continents, specialists follow different treatment concepts, e.g., involving the discussion about efficacy of betahistine and diuretics [7, 8], debating when ablative or non-ablative options should be introduced, and the widely debated effect of the non-ablative endolymphatic sac surgery (ESS) [9]. However, a current international consented recommendation provides a staged treatment concept considering non-ablative and ablative treatment options as the following [10]: (1) conservative medical treatment with lifestyle and diet adjustments, vestibular rehabilitation, and betahistine orally [11–15]; (2) intratympanic injections of corticosteroids [16]; (3) ESS [17]; (4) as the first ablative option intratympanic injections of the ototoxic antibiotic gentamicin [18]; and (5) as *ultima ratio* the labyrinthectomy [19]. Since the 1920s the idea of manipulation on the inner ear such as decompressing the endolymphatic sac evolved when Portmann drew parallels to glaucoma [20] which was later expanded by William House in the 1960s by inserting a permanent shunt into the subarachnoid space or mastoid [21]. The history of establishing different techniques on surgical manipulation of the inner ear for treatment of MD is depicted in the review of Kersbergen and colleagues [22]. Different methods have been evaluated to date, from performing a mere decompression of the endolymphatic sac, an endolymphatic sac incision, an endolymph-mastoid shunt surgery with insertion of a small silicone shunt, to the most invasive technique, the endolymph-subarachnoid space shunt [17, 21, 23]. However, contradictory prevalence data on radiological temporal bone specifics exists suggesting a potential hypoplasia or degeneration of the vestibular duct and endolymphatic sac [24–27]. Thus, efficacy of ESS with a hypoplastic or degenerated endolymphatic sac is called into question. In addition, clinical efficacy on this treatment option is widely debated as ESS holds a common perception of a placebo surgery as stated in the early work of Thomsen et al. [9, 28–35]. Many other, solely retrospective studies, report of a high rate of vertigo control postinterventional [17] and a non-ablative character of this treatment option. A recent meta-analysis of Szott and colleagues revealed a mean hearing impairment postoperatively of a pure tone average of around 9 dB and 25% speech discrimination [36]. Thus, further assessment of ESS with regard to efficacy, quality of life and postoperative hearing is demanded. Volume and the complex regulation of its inner ear fluid compositions [37] could be altered by manipulation on the endolymphatic sac by ESS. However, effect of opening the perilymphatic system has not been evaluated systematically in MD patients so far. In cochlear implantation (CI), the perilymphatic system is routinely opened by inserting the electrode via the round window. Due to the quality-of-life impairing effect of episodic vertigo attacks, all treatment concepts focus on the reduction of these attacks and less so on hearing rehabilitation. Moreover, fitting acoustic hearing aids in those patients who might intermittently experience fluctuating hearing levels is challenging and often demotivating, especially when the contralateral ear is healthy with normacusis. Therefore, in the present study, we (1) investigated the potential effect on reduction of vertigo by alternating the pressure within the endo- and in addition perilymphatic system by comparing patients with ESS and/or CI surgery and (2) evaluated hearing rehabilitation results in these patient groups.

## Materials and methods

### Patient selection and ethical considerations

We performed a retrospective data analysis, which was approved on April 11th, 2019 by the Institutional Review Board of the University Hospital, LMU Munich (Ethikkommission der Medizinischen Fakultät der LMU München), reference number 19–086. Demographic data (gender, age, date of surgery, date of last visit, past medical history, pre- and postoperative treatment, description of vertigo, presence, and grading of an endolymphatic hydrops in the MRI) was collected from the electronic clinical patient database. In total, 141 consecutive patients with the diagnosis Menière’s disease who were treated at a tertiary center from 2004 to 2020 with ESS alone, CI alone, or ESS plus CI were identified. Patients who did not meet the diagnostic criteria of at least definitive or probable MD according to Lopez Escamez et al. [5] or whose data were not sufficient to apply these diagnostic criteria (*n = *55) were excluded, as well as patients who did not suffer from vertigo attacks at the time of surgery. The remaining 86 patients with unilateral definitive (dMD) or probable MD (pMD) were divided into the groups: “endolymphathic sac surgery alone” (ESS^alone^), “cochlea implantation alone” (CI^alone^), and “endolymphathic sac surgery and cochlea implantation” (ESS + CI). The group of ESS + CI included patients who received ESS with CI simultaneously and those who received both procedures consecutively. ESS was performed as either endolymphatic sac decompression or as an incision of the endolymphatic sac with inserting a small triangular silicone foil resulting in an endolymph-mastoid shunt.

### Surgical procedures: endolymphatic sac surgery and cochlear implantation

For all patients of the group ESS^alone^, a standard endolymphatic mastoid shunt surgery (EMSS) was performed as described previously [17]. Briefly, a mastoidectomy with blue lining of the posterior semicircular canal was performed, then the skull base with demonstrating the endolymphatic sac (ES) as a duplicature of the dura mater dissected, the ES incised and a triangular silicone foil as a shunt inserted (edge length 2 × 3 mm). For the CI^alone^ group, a standard CI-surgery via the round window was performed with implanting either a cochlear implant from the company Cochlear (Cochlear Ltd., Sydney, Australia) or MED EL (MED-EL GmbH, Innsbruck, Austria). Patients of the ESS + CI received either EMSS with CI surgery simultaneously, or first a EMSS or endolymphatic sac decompression surgery and then CI surgery.

### Postoperative management

Postoperatively, all patients received regular otologic follow-up including audiometric testing of bone conduction, as well as monitoring for facial nerve impairment or nystagmus. CI patients received the first fitting of the speech processor 4–6 weeks postoperatively. In the first year after CI surgery, fittings were routinely performed at 1, 3, 6, 9, and 12 months after first activation, which is performed 4 weeks postoperatively, and then at least once a year.

### Vestibular function

Documentation of vestibular testing pre- and postoperatively was available in only a share of patients. Since caloric testing and/or video head impulse test was not consistently documented, or in some cases only “adequate peripheral vestibular function” or “unilateral vestibulopathy” (UVP) or “bilateral vestibulopathy” (BVP), no absolute or relative values were analyzed.

Regarding evaluation of pre- and postoperative vertigo attacks, patients’ symptom diaries and doctor’s notes from the hospitals data base were evaluated. In cases with rather undetailed documentation the judgement of the treating physicians whether further MD treatment escalation was indicated, such as increasing betahistine dosage, intratympanic corticosteroid injections, or proceeding to endolymphatic sac surgery was considered and thus, the documented vertigo was classified as vertigo attacks resulting from the MD.

### Pure tone audiometry

Audiometric tests were performed as a pure tone audiometry with testing for each ear separately at 0.250, 0.5, 1, 2, 3, 4, and 6 kHz via headphones and with air and bone conduction thresholds between -10 dB and 120 dB hearing level (dB HL). Aided air conduction was performed with warble tones in free-field. For statistical purposes, thresholds exceeding 120 dB HL were recorded as 130 dB HL.

### Speech audiometry

Regarding speech discrimination of monosyllabic words in quiet, the German language Freiburg Monosyllabic Test was used as described before [38]. In brief, 20 monosyllabic words recorded from a male speaker were presented to the patients. Each correctly recognized word was accounted for 5%, the maximum score is 100%. Speech discrimination rate was measured in quiet at 65 dB HL. Then, the optimal fitted volume in dB was evaluated and the maximal percentage of discriminated monosyllables was documented (= dB-opt). Timepoint of evaluation was for the ESS^alone^ group not earlier than 1 month and for ESS + CI and CI^alone^ around 1 year after receiving the CI.

### Data analysis

For preoperative analysis the last available results prior to surgery were considered. Regarding postoperative analysis for ESS^alone^, the earliest postoperative available data on speech perception was analyzed. For any CI-patient, available data after at least 12 months post-implantation were accounted for analysis.

### Statistical analysis

Solely descriptive analysis was performed. All figures were created with Microsoft Excel version 16.64 for Mac OS.

## Results

### Demography and preoperative Menière’s disease treatment

After applying inclusion and exclusion criteria, 86 patients were eligible for further analysis. The patients were assigned to the following 3 groups: ESS^alone^, CI ^alone^, and ESS + CI. Regarding age, mean values of the CI^alone^ (64.2 ± 11.4 years) and ESS + CI (63.1 ± 9.7 years) groups were similar. Mean age of the ESS^alone^ was substantially younger with 56.2 ± 13.0 years. Gender was evenly distributed over all groups. Regarding the categories according to diagnostic criteria, the share of patients with probable MD was the largest in the ESS^alone^ group (*n = *19, 40.4%), followed by ESS + CI group (*n = *6; 21.7%), whereas the CI^alone^ group only included patients with definitive MD. Presence of an endolymphatic hydrops in the MRI and preoperative Menière specific treatment did not show any trend toward a difference. Intratympanic application of steroids was seldomly performed prior to ESS or CI surgery. Thus, only 1 patient in the group ESS + CI and 2 patients in ESS^alone^ received intratympanic steroid injections. Regarding ablative MD treatment, 1 patient in group ESS + CI and 2 patients in ESS^alone^ received intratympanic gentamicin injections. Further documentation as to why this course of treatment was chosen was not apparent from the chart notes. An overview is given in Table 1.

**Table 1:** Patients’ characteristics

	ESS^alone^	CI^alone^	ESS + CI
Number	45	12	29
Age (years ± SD)	56.2 ± 13.0	64.2 ± 11.4	63.1 ± 9.7
Gender (%)	24 male (53.5%)	6 male (50.0%)	17 male (58.6%)
Side (%)	25 (right)	6 (right)	12 (right)
MD category^a^			
Definitve MD (%)	28 (59.6)	12 (100.0)	23 (79.3)
Probable MD (%)	19 (40.4)	0	6 (21.7)
Endolymphatic hydrops in MRI			
None	1	0	2
I	1	2	3
II	16	1	6
III	3	0	1
Unknown	26	9	15
Preoperative treatment			
None	5	0	3
Betahistine 12 mg TID	2	1	1
Betahistine 24 mg TID	17	5	7
Betahistine 48 mg TID	10	3	7
Betahistine 72 mg TID	4	1	2
Betahistine 96 mg TID	3	0	1
Steroids i.t	2	0	1
Gentamicin i.t	2	0	1
Unknown	2	2	7

Patient’s characteristics including pre- and postoperative additional Meniére specific treatment and share of endolymphatic hydrops depicted in MRI

*CI*^*alone*^ group of patients who received a cochlear implantation alone; *ESS* endolymphatic sac surgery; *ESS*^*alone*^ group of patients who received an endolymphatic sac surgery alone; *ESS + CI* group of patients who received an endolymphatic sac surgery and cochlear implantation; *i.t.* intratympanic application; *MD* Menière’s disease; *SD* standard deviation; *TID* *ter in die*; *yr* years

^a^According to Lopez-Escamez et al. [5]

### Perioperative data, outcome and postoperative Menière’s disease treatment

Relieve of symptoms in terms of improvement of vertigo control was observed in all three groups and the highest rate achieved in all CI^alone^ patients, followed by the ESS + CI (*n = *26; 89.7%) and the ESS^alone^ group (*n = *31; 66.0%). Regarding patients who received ESS, for the ESS^alone^ group all patients and of the ESS + CI group the larger share (*n = *25; 86.2%) received an EMSS with insertion of a small silicone shunt. The reason why in 4 ESS + CI patients an ES decompression was performed was not documented. Postoperative MD specific treatment was never required in CI^alone^ patients. By contrast, 2 ESS + CI patients (6.9%) required postoperative ablative treatment with intratympanic gentamicin injections and 6 ESS^alone^ patients (13.3%) of whom 5 were ablatively treated with intratympanic gentamicin injections and 1 with a labyrinthectomy. All data is shown in Table 2.

**Table 2:** Treatment and outcome

	ESS^alone^	CI^alone^	ESS + CI
Number	45	12	29
Type of ESS			
ES-decompression	0	n.a	4
EMSS with silicone	47	n.a	25
Length of CI electrode			
Cochlear 422^a^	n.a	2	0
Slim Straight^a^	n.a	1	2
Flex 28^b^	n.a	2	23
Flex soft^b^	n.a	4	3
Standard^b^	n.a	3	0
Vertigo improvement	31 (66.0)	12 (100.0)	26 (89.7)
Postoperative treatment			
None	33	12	27
Betahistine 12 mg TID	0	0	0
Betahistine 24 mg TID	5	0	0
Betahistine 48 mg TID	2	0	0
Betahistine 72 mg TID	1	0	0
Steroids i.t	1	0	0
Gentamicin i.t	5	0	2
Revision EMSS	4	n.a	0
Labyrinthectomy	1	0	0

Treatment, treatment outcome, and postoperative treatment of all 86 patients with Morbus Menière presented in the three groups endolymphatic sac surgery with CI, endolymphatic sac surgery alone, and CI alone

*CI* cochlear implant; *CI*^*alone*^ group of patients who received a cochlear implantation alone; *EMSS* endolymphatic mastoid shunt surgery; *ES* endolymphatic sac; *ESS* endolymphatic sac surgery; *ESS*^*alone*^ group of patients who received an endolymphatic sac surgery alone; *ESS + CI* group of patients who received an endolymphatic sac surgery and cochlear implantation; *i.t.* intratympanic; *n.a.* not applicable; *TID* *ter in die*

^a^Implant from the company Cochlear Germany GmbH & Co. KG

^b^Implant from the company MED EL GmbH, Innsbruck, Austria

### Vestibular function

Pre- and postoperative peripheral vestibular function was available in only a share of all patients. Overall, the CI^alone^ patients accounted for the largest share of patients with UVP/BVP preoperatively (*n = *6; 50.0%), followed by ESS + CI patients (*n = *11; 37.9%). The best function preoperatively was seen in the ESS^alone^ group (88.9%; *n = *24/27 no peripheral vestibular impairment). Postoperatively, in 3 of those 24 patients (12.5%) an UVP was evident (Table 3).

**Table 3:** Pre- and postoperative hearing and vestibular function

	ESS^alone^	CI^alone^	ESS + CI
Pure tone audiometry (*n*^subset^/total)	34/45	10/12	26/29
Preoperative			
AC-PTA^7^ unaided (dB HL ± SD)	62.2 ± 10.6	96.5 ± 14.6	90.5 ± 16.9
BC-PTA^7^ unaided (dB HL ± SD)	60.1 ± 13.2	90.5 ± 10.0	83.4 ± 9.0
Postoperative			
AC-PTA^7^ unaided (dB HL ± SD)	69.7 ± 11.1	n.a	n.a
BC-PTA^7^ unaided (dB HL ± SD)	64.3 ± 17.2	n.a	n.a
PTA^7^ aided (dB HL ± SD)	n.a	42.1 ± 6.1	45.8 ± 7.1
Vestibular function			
Preoperative			
Preoperative UVP	3	4	8
Preoperative BVP	0	2	3
Postoperative			
No change	13	0	0
Worsen	6	0	1
Unknown	27	6	17

Results of pure tone audiometry and vestibular function

*AC* air conduction; *BC* bone conduction; *BVP* bilateral vestibulopathiy; *CI*^*alone*^ group of patients who received a cochlear implantation alone; *dB HL* dezible hearing level; *ESS*^*alone*^ group of patients who received an endolymphatic sac surgery alone; *ESS + CI* group of patients who received an endolymphatic sac surgery and cochlear implantation; *n* number; *PTA*^*7*^ pure tone average of the frequencies 0.250, 0.5, 1, 2, 3, 4, 6 kHz; *SD* standard deviation

### Pure tone audiometry

Audiometric testing was available in a subset of patients and showed overall better preoperative values for the ESS^alone^ group (Table 3). Regarding postoperative results, 12 months after surgery the ESS^alone^ performed the worst (ESS^alone^ AC-PTA^7^ unaided 69.7 ± 11.1 dB HL vs. PTA^7^ aided CI^alone^ 42.1 ± 6.1 dB HL and ESS + CI 45.8 ± 7.1 dB HL).

### Speech audiometry

**Fig. 1:**
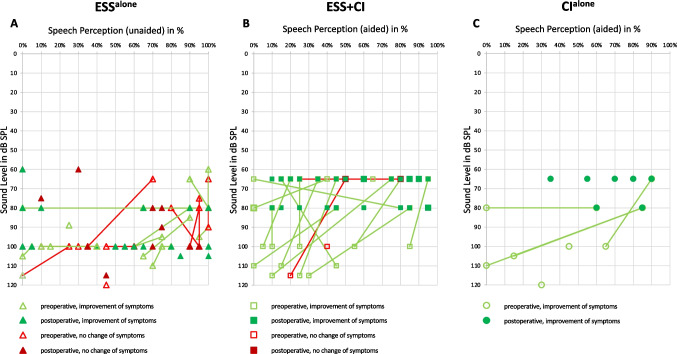
Speech Perception with Freiburg Monosyllabic Test. Best monosyllabic speech perception with Freiburg monosyllabic test in percent (x-axis) and respective necessary volume in dB (y-axis) are depicted pre- (empty lighter symbols) and postoperatively (darker filled symbols). Available data of every patient is represented by one symbol and pre- and postoperative values are connected by a line. Patients with vertigo control are marked green, patients with no improvement of results red. **A** Shows patients after endolymphatic sac surgery alone (ESS^alone^, triangles; preoperative *n = *34; postoperative *n = *34), **B** patients with cochlear implantation alone (CI^alone^, circles; preoperative *n = *6; postoperative *n = *10), and **C** patients with endolymphatic sac surgery plus cochlear implantation (ESS + CI, squares; preoperative *n = *18; postoperative *n = *24)

Regarding speech perception with the Freiburg Monosyllabic Test, data was available in only a subset of patients. Preoperative performance seemed similar in all three groups (Fig. 1). The postoperative performance was the worst in the group of ESS^alone^ (pre- and postoperative data available in *n = *34, range 1–19 months postoperatively) and comparably improved in both groups with CI patients (CI^alone^ preoperative *n = *6; postoperative *n = *10, range 13–37 months postoperatively; ESS + CI: preoperative *n = *18; postoperative *n = *24, range 13–52 months postoperatively). All data is depicted as scattergrams with pre- and postoperative data and enhancement of vertigo improvement in Fig. 1.

## Discussion

The study at hand investigated the effect of three different treatment variants on vertigo control in patients with MD: ESS, CI, and patients who received both. Endolymphatic hydrops is a widely discussed and assumed epiphenomenon in MD patients [1]. However, the effect on the endolymphatic hydrops by opening the endolymphatic sac by ESS is debatable, since patients with MD might have a hypoplastic or degenerated endolymphatic sac [24]. The rationale behind this study was to investigate a potential effect of manipulation on the perilymphatic system during cochlea implantation in comparison with or as an additive effect of the manipulation on the endolymphatic system with ESS.

The results of the present study acknowledge an improvement of vertigo in all three groups; however, the best vertigo control rate in patients treated with CI^alone^, followed by patients with ESS + CI. In comparison, those with ESS^alone^ had the worst symptom reduction rate. Interestingly, regarding MD categories, the group CI^alone^ had the largest share of patients with definitive MD, followed by ESS + CI, and ESS^alone^ had the lowest share. Thus, distribution of the MD categories might have an influence on the treatment success, which has not been investigated so far to the best of our knowledge. In addition, one can discuss if those patients with CI indications might possibly represent a final stage of MD. Another confounder should be considered as well: since in the present study pre- and postoperative data on the vestibular function is available only in a share of cases, evaluation of vertigo control is based on subjective parameters read from the patient chart. When looking at the scarce data on vestibular function in the present study, the larger share of patients with preoperative vestibulopathy in both CI groups is noticeable, as well as a larger share of adequate postoperative vestibular function in the ESS^alone^ group, but still not enough to draw a sound conclusion. Regarding hearing results, we observed similar preoperative values in all three groups and substantially better hearing postoperatively in the CI rehabilitated patients. This underlines once again the importance of hearing counselling for MD patients in general, for whom hearing loss does not seem to contribute to impairment of quality of life as much as the vertigo attacks. One reason might be the single sided deafness situation, with which the level of suffering seems not to matter as much as the impairment due to the vertigo attacks. To our knowledge, this again is an issue that has not been evaluated properly to date and the influence of hearing loss and/or vertigo on quality of life remains to be investigated. Counselling the patient properly and illustrate the benefit of binaural hearing and improved speech perception, the potential effect on vertigo control may be an additional argument.

Limitations of this study lie in the retrospective characteristics with limited subject numbers of the different groups, incomplete data on pre- and postoperative vestibular function, as well as lack of standardized vertigo documentation pre- and postoperatively, e.g., a vertigo diary or vertigo questionnaires. Moreover, a fair share of cases had to be excluded from the analysis, because the diagnostic criteria were not fulfilled in those patients, or documentation on the vertigo history was insufficient to apply the diagnostic criteria at all. On the upside, the present study is the first to our knowledge to launch the discussion on a potential influence of manipulating the endolymphatic versus the perilymphatic system in a clinical setting. Other studies evaluated the effect on ESS alone (see broad literature table in [17] or on CI with or without labyrinthectomy [39].

In general, ESS is a well-discussed treatment option for MD and has been studied widely, with a successful reduction of vertigo attacks and stable postoperative cochlear and vestibular function [17]. Different surgical techniques are offered, ranging from a simple decompression of the endolymphatic sac to the most invasive procedure of creating a shunt between the endolymphatic sac and the subarachnoidal space [21]. The most commonly applied technique is an incision of the endolymphatic sac and creation of a permanent shunt by inserting a small silicon foil [17, 36]. The effect on alteration of vertigo attacks of additionally opening the perilymphatic system is unclear. With performing cochlear implantation, the perilymphatic system is temporarily opened via the round window. When the electrode is inserted into the scala tympani, perilymphatic fluid is automatically pressed out, and thus, alterations within the perilymphatic systems are possible. In addition, especially with longer and thicker electrodes, the scala tympani is compressed and a lesion of the scalae cannot be ruled out in those cases.

The effect of CI surgery in general on the vestibular system has been investigated thoroughly [40, 41]. A recent meta-analysis reviewing 30 studies found significant alterations of vestibular evoked myogenic potentials (VEMP) and caloric results after cochlear implantation [41]. These findings are supported by a study performed in 2015 by Kuang et al. [42] and a different meta-analysis from 2017 [40]. From an anatomical perspective, this seems comprehensible, considering that the saccule and utricle lie next to the round window, however, within the endolymphatic and not perilymphatic system. Nevertheless, results of a vertigo-related quality of life questionnaire (*Dizziness Handicap Inventory*—DHI), head impulse test (HIT), video-HIT, and posturography seemed to remain unaltered after cochlear implantation [40, 41]. When looking at vertigo improvement in MD patients, literature reveals only one systematic review, which investigated 37 studies [39]. In these 37 studies, 216 patients with MD were identified, thereof 84 with unilateral and 119 with bilateral MD. Of those, 172 received a CI without labyrinthectomy and reported an overall improvement of vertigo symptoms given by the results of the DHI. However, comparing this review with the present study, data on MD categories, stages of MD, and postoperative vestibular function were not mentioned [39].

## Conclusion

Data from the current study showed vertigo improvement after both ESS and CI. However, it suggests a beneficial effect of CI surgery in comparison with ESS regarding vertigo control, potentially contributed by the manipulation of both the endo- and perilymphatic system. Whether this is contributed by the deterioration of vestibular hair cells due to the manipulation and trauma within the inner ear or whether there is a true treatment effect cannot be answered based on the data of the present study. A more systematic characterization of the patients with larger case numbers and documentation of follow up data would be needed to evaluate a clinical effect more properly.

## Data Availability

Original data are available on demand.

## Code availability

Solely descriptive analysis was performed. All figures were created with Microsoft Excel version 1906.
